# Supplementary material for: Do nutritional interventions before or during pregnancy affect placental phenotype? Findings from a systematic review of human clinical trials
Source: J Glob Health. 2024 Dec 20;14:04240. doi: 10.7189/jogh.14.04240 (PMC11658718; doi:10.7189/jogh.14.04240)
Supplement: Online Supplementary Document [file jogh-14-04240-s001.pdf]

Online Supplementary Material

Supplementary Methods	Pages 2-4
Supplementary Results	Pages 5-10
Supplementary Figures	Page 11
Supplementary Tables	Pages 12-38

Supplementary Methods

Information sources and search terms

PubMed, ClinicalTrials.gov, and the World Health Organization (WHO) International Clinical Trials Registry Platform (ICTRP) were searched to identify peer-reviewed publications using the following search string: ((nutr* OR diet* OR supplement* OR vitamin OR folate* OR folic acid OR mineral OR (micronutri* AND (intervention OR supplement*))) AND (placenta* OR preeclampsia) AND (maternal OR pregnan* OR gravid* OR conception* OR preconceptional OR gestation*)). When searching the listed databases, the filters "Clinical Study, Clinical Trial, Comparative Study, Controlled Clinical Trial, Journal Article, Humans" were applied.

Article search, screening, and data collection

A three-level screening process was performed by two authors (VB and MW; Figure 1). A total of 5299 titles from PubMed (n=5078), ClinicalTrials.gov (n=156), and WHO ICTRP (n=65) were captured from the article search. Articles were excluded for deduplication (n=14), publication date before 2001 (n=8), and incorrect study type (n=10), leaving 5267 articles to be screened at level one (title screening). Clinical trial titles from ClinicalTrials.gov and WHO ICTRP, and article titles from PubMed search results were screened for relevance to our review objectives. In total, 4568 titles were excluded for having a topic not relevant to this review, leaving a total of 699 article abstracts and clinical trials documents to be screened at level two.

At screening level two, 699 article abstracts (PubMed: n=608) and clinical trial records (ClinicalTrials.gov: n=60, WHO ICTRP: n=31) were reviewed to determine their adherence to the inclusion criteria (Figure 1). Articles and trials were excluded for: not reporting on a direct maternal nutritional intervention (n=323), reporting on an animal study (n=12), incorrect type of article (i.e., review, study protocol, or commentary; n=169), no results posted (n=29), or not written in English (n=2), leaving 164 records (PubMed articles: n=129, ClinicalTrials.gov: n=17, WHO ICTRP: n=18) to be carried forward to level three screening.

At level three, full texts were unavailable for seven of 129 PubMed articles. The corresponding authors were contacted by email twice between December 2021 and January 2022 for each of these seven articles, but only one response was received. Publications resulting from the clinical trials retained for screening at level three were obtained from the trial webpage directly, or from searching the trial name and/or number using search browsers (e.g., Google Scholar). Ultimately, 158 full texts were reviewed to evaluate whether studies met the full inclusion criteria (from PubMed, n=123; ClinicalTrials.gov, n=17, and WHO ICTRP, n=18; Figure 1). At level three, articles were excluded for not including a placental measure (n=64; including articles reporting on relationships between a nutritional intervention and risk of preeclampsia that did not provide placenta-specific measures), not administering a direct nutritional intervention (n=9), duplicates (n=11), or other reasons (i.e., wrong study type, no trial results available or could not be found (n=21). In total, 53 articles met the full inclusion criteria.

Data extraction

Data were extracted from each of the 53 articles, including population data (such as study location [which we used to identify socioeconomic status based on four World Bank classifications: low-income (LIC), low-middle income (LMIC), upper-middle income (UMIC), and high-income

(HIC)] (1)), maternal clinical characteristics and demographics, type of pregnancy (singleton or twin), and nutritional intervention details (type [micronutrient, lipid, protein, and/or diet- or lifestyle-based], composition, timing, dose, and compliance data). Data on maternal comorbidities (defined in this study as conditions or states of health during pregnancy that may be harmful to the health of the mother and/or fetus) reported in the studies under review were noted and used to inform results interpretations. Key results from each individual study were extracted and summarized, and the proportion of studies that did or did not report placental changes or improvements in maternal and offspring outcomes was calculated for each subtype of nutritional intervention (micronutrient, macronutrient, and diet and lifestyle-based). Reported adverse outcomes were noted and classified into two categories: adverse effects (outcomes that were suspected to be in response to the intervention (2)) and adverse events (outcomes that were not suspected to be in response to the intervention (2)). Data on associations between the nutritional intervention and 1. placental phenotype (primary outcome), and 2. placental sex, and 3. fetal/infant and maternal outcomes (secondary outcomes) were also captured.

Risk of bias assessments

The articles under review were assessed for risk of bias (RoB) using the Cochrane Collaboration's Tool for Assessing Risk of Bias for randomised studies (n=50; Supplementary Table S3) and the Risk Of Bias In Non-randomised Studies of Interventions (ROBINS-I) tool for non-randomised studies (n=3) (3, 4) .The RoB assessment criteria were set *a priori*. As our outcome of interest was placental phenotype, which was often not the primary outcome of the studies under review, assessments for risk of bias due to "incomplete outcome data" considered (when applicable) how the subset of the original cohort with placental data was selected or determined. We also assessed potential bias related to compliance to the intervention, as follows: articles that clearly measured and reported participant compliance were assessed as low risk, articles that did not report clear methods to measure compliance, but had frequent follow up visits with participants, were assessed as unclear risk, and articles that did not measure compliance and had few participant follow up visits (e.g., only at intervention onset and delivery) were assessed as high risk. RoB assessments were performed independently by two authors (VB and MW). Discrepancies in RoB assessments were resolved through discussion with a third author (KLC). While RoB in interventions was not a primary outcome of this review, assessments of RoB were performed to inform results interpretations.

Data synthesis and visualisation

Graphical Overview for Evidence Reviews (GOfER (5)) figures were created for each type of nutritional intervention to visualise key study data. The GOfER included intervention composition and timing, reported alterations to placental phenotype, and reported maternal and fetal outcomes (both beneficial and adverse). A filled map was created to demonstrate the locations of the studies included (Microsoft Excel v16). Alluvial diagrams were created to visualise relationships between type of intervention, study location, and changes in placental phenotype (or no changes; RAWGraphs (6)).

References

1. World Bank Group. Country Classification: World Bank Country and Lending Groups: The World Bank; 2022.
2. Sheyholislami H, Connor KL. Are Probiotics and Prebiotics Safe for Use during Pregnancy and Lactation? A Systematic Review and Meta-Analysis. Nutrients. 2021;13(7). Epub 20210713. doi: 10.3390/nu13072382. PubMed PMID: 34371892; PubMed Central PMCID: PMC8308823.
3. Higgins JPT, Altman DG, Gøtzsche PC, Jüni P, Moher D, Oxman AD, et al. The Cochrane Collaboration's tool for assessing risk of bias in randomised trials. BMJ. 2011;343:d5928. doi: 10.1136/bmj.d5928.
4. Sterne JA, Hernán MA, Reeves BC, Savović J, Berkman ND, Viswanathan M, et al. ROBINS-I: a tool for assessing risk of bias in non-randomised studies of interventions. BMJ. 2016;355:i4919. Epub 20161012. doi: 10.1136/bmj.i4919. PubMed PMID: 27733354; PubMed Central PMCID: PMC5062054.
5. Chu J, Hardy P, Beeson L, Coomarasamy A. What is the best method for managing early miscarriage? BMJ. 2020;368:l6438. Epub 20200120. doi: 10.1136/bmj.l6438. PubMed PMID: 31959625.
6. Mauri M, Elli T, Caviglia G, Uboldi G, Azzi M. RAWGraphs: A Visualisation Platform to Create Open Outputs. Proceedings of the 12th Biannual Conference on Italian SIGCHI Chapter; Cagliari, Italy: Association for Computing Machinery; 2017. p. Article 28.

Supplementary Results

Study and cohort characteristics

The majority (n=46 [87%]) of studies included only singleton pregnancies, while seven (13%) included both singleton and twin pregnancies. Maternal age in the included studies ranged from 15 to 45 years old, with most studies reporting an average maternal age in the mid- to late twenties. Five studies enrolled mothers under the age of 18 (1-5). Maternal comorbidities were common in the studies under review (n=33 [62%]) and were either a criterion to be a participant in the nutritional intervention trial or were identified as a potential confounding variable by the original authors. The most common comorbidities included a history or diagnosis of preeclampsia (n=12 [36%]), prior or current hypertension (n=7 [21%]), overweight and/or obesity (n=7 [21%]), smoking (n=6 [18%]), and/or any type of diabetes (n=6 [18%]; Supplementary Table S4). Twenty studies excluded pregnant participants with comorbidities.

Presence of a maternal nutrient deficiency was a selection criterion for three (5.6%) of the studies. All studies reported self-administration of the intervention by the participant, and three studies also included an additional intervention administered by a research team member once weekly. Interventions began either peri- (n=3 [6%]) or post-conceptionally (n=50 [94%]; 14 beginning in the first trimester, 28 in the second trimester, and 11 in the third trimester), with the majority continuing until birth (n=52 [98%]), and one that finished after 12 weeks of administration.

Adverse effects and events were reported in five and one study, respectively, and included gastrointestinal complaints following multiple micronutrient (3) or iron (6) supplementation, an increased incidence of preterm birth following iron supplementation (1), an increased likelihood of antenatal hospital admission with hypertension following vitamin C+E supplementation (exact cause unknown to the researchers) (7), and increased PROM risk and reports of general developmental abnormalities in both the treatment vitamin C+E and placebo groups (8).

Nutritional interventions associated with improved maternal and infant outcomes

There were 13 nutritional interventions that associated with improved maternal outcomes, including one diet and lifestyle-based intervention, one protein-based intervention, four lipid-based interventions, and seven micronutrient-based interventions (Figure 5A). Improved maternal outcomes included a smaller required insulin dose in mothers with gestational diabetes (following vitamin C supplementation (9)), reduced blood levels of iron and/or zinc (following iron (10) and multiple micronutrient supplementation (11)), decreased total lipid content in placental tissue (following docosahexaenoic acid [DHA] and eicosapentaenoic acid [EPA] supplementation (12)), decreased risk of preeclampsia (following calcium (13), L-arginine and multiple micronutrient supplementation (14)), and decreased risk of placental abruption (following magnesium citrate supplementation (15); Figure 5A).

Of the seven nutritional interventions that associated with improved infant outcomes, two were diet and lifestyle-based, one was protein-based, two were lipid-based, and two were micronutrient-based (Figure 5B). Improved infant outcomes included improved fetal growth (following lipid supplementation (16)), increased fetal nervonic acid ratio (following EPA and DHA supplementation (17)), and a decreased risk of intrauterine growth restriction (IUGR; following L-arginine supplementation (18)), twin-to-twin transfusion syndrome incidence (TTTS [a syndrome

that can occur in diamniotic monozygotic twin pregnancies that creates an imbalance in nutrient allocation between fetuses]; following Ensure® liquid supplementation (19)), and preterm birth (for infants born to mothers who smoked; following vitamins C+E (supplementation with both vitamins concurrently) (20); Figure 5B).

Interventions associated with placental phenotype are more likely, overall, to improve maternal and offspring outcomes

Maternal outcomes positively associate with placental changes

Of the eight micronutrient-based intervention studies that reported improved maternal outcomes, seven (89%) reported associations between the intervention and placental phenotype. Placental changes and improved maternal outcomes included: a reduction in oxidative stress markers in both the placental tissue and maternal blood plasma (following vitamin C supplementation starting once the mother was diagnosed with gestational diabetes (9)) and increased mRNA expression of placental iron uptake transferrin receptor 1 and increased maternal iron and zinc levels (after multiple micronutrient supplementation initiated in the second trimester (11)), and decreased risk of placental abruption (which was classified as both a placental and maternal outcome in this study; following magnesium citrate supplementation starting in the second trimester (15)). Additionally, reduced risk of preeclampsia was reported in four studies, as discussed above (13, 14, 21, 22).

For macronutrient-based interventions, all four of the lipid-based studies that reported improved maternal outcomes also reported placental changes following nutritional intervention. Omega-3 supplementation beginning in the first trimester was associated with increased maternal plasma DHA and EPA levels and decreased expression of placental factors like interleukin 6 and 8 (23), whilst omega-3 supplementation beginning in the second trimester was associated with increased maternal plasma DHA and EPA enrichment, circulating maternal DHA and EPA (73), and decreased total lipid content and ability of the placenta to store and esterify lipids (12). Further, daily fish oil supplementation initiated during the third trimester was associated with increased maternal circulating DHA and plasma nervonic acid content and increased fatty acid concentration in placental tissue (17). Reduced risk of preeclampsia, but not placental abruption, was reported in one protein-based study as discussed above (14).

One of the four diet and lifestyle-based interventions that included maternal outcome data reported improved maternal outcomes following intervention without changes in the placenta (24).

Offspring outcomes positively associate with placental changes

Of the 12 micronutrient-based interventions that included data on offspring outcomes, two (17%) reported changes in placental phenotype and improved offspring outcomes following nutritional intervention. In mothers with gestational diabetes mellitus, daily supplementation with vitamin C from diagnosis onwards was associated with a reduction in both fetal and placental oxidative stress markers and newborn birthweight, in comparison to the non-treatment group (9). A decreased risk of both preterm birth and placental abruption was also reported in a cohort of mothers who smoked during pregnancy following daily vitamins C+E supplementation initiated during the first trimester (20). One of the 12 studies with offspring outcome data also reported an adverse offspring outcome

(increased incidence of preterm birth) following daily ferrous gluconate supplementation from preconception to birth, despite noting a reduced risk of chorioamnionitis (1).

Of the macronutrient-based interventions, two of four lipid-based interventions that reported offspring outcome data observed altered placental phenotype and improved offspring outcomes. SQLNS supplementation initiated preconceptionally was associated with improved fetal growth, increased placental area (16), and changes in expression of multiple placental metabolic genes. Fish oil supplementation initiated in the third trimester was associated with higher offspring plasma nervonic acid content, increased polyunsaturated placental fatty acid composition, but no changes to visual or cognitive development outcomes measured in the offspring (17). Additionally, two protein-based interventions reported on offspring outcomes, with one noting increased cerebro-placental ratio and decreased incidence of intrauterine growth restriction (IUGR) following daily L-arginine supplementation initiated during the third trimester (18).

Improved offspring outcomes were reported in two diet and lifestyle-based intervention studies, one of which also reported placental changes following the intervention. First, Ensure® liquid supplementation initiated upon diagnosis of a monochorionic diamniotic pregnancy was associated with decreased risk of TTTS and incidence at delivery time (19). Second, daily chlorella supplementation beginning in the second trimester was associated with decreased fetal dioxin transfer (measured through decreased total toxic equivalents), but no significant change in toxin concentrations pre- and post-treatment (25). Notably, we determined the second study to have a high RoB.

References

1. Brabin B, Gies S, Roberts SA, Diallo S, Lompo OM, Kazienga A, et al. Excess risk of preterm birth with periconceptional iron supplementation in a malaria endemic area: analysis of secondary data on birth outcomes in a double blind randomized controlled safety trial in Burkina Faso. Malar J. 2019;18(1):161. Epub 20190506. doi: 10.1186/s12936-019-2797-8. PubMed PMID: 31060615; PubMed Central PMCID: PMC6501288.
2. Ormesher L, Myers JE, Chmiel C, Wareing M, Greenwood SL, Tropea T, et al. Effects of dietary nitrate supplementation, from beetroot juice, on blood pressure in hypertensive pregnant women: A randomised, double-blind, placebo-controlled feasibility trial. Nitric Oxide. 2018;80:37-44. Epub 20180809. doi: 10.1016/j.niox.2018.08.004. PubMed PMID: 30099096.
3. Owens S, Gulati R, Fulford AJ, Sosseh F, Denison FC, Brabin BJ, et al. Periconceptional multiple-micronutrient supplementation and placental function in rural Gambian women: a double-blind, randomized, placebo-controlled trial. Am J Clin Nutr. 2015;102(6):1450-9. Epub 20151111. doi: 10.3945/ajcn.113.072413. PubMed PMID: 26561613; PubMed Central PMCID: PMC4658455.
4. Kiondo P, Wamuyu-Maina G, Wandabwa J, Bimenya GS, Tumwesigye NM, Okong P. The effects of vitamin C supplementation on pre-eclampsia in Mulago Hospital, Kampala, Uganda: a randomized placebo controlled clinical trial. BMC Pregnancy Childbirth. 2014;14:283. Epub 20140821. doi: 10.1186/1471-2393-14-283. PubMed PMID: 25142305; PubMed Central PMCID: PMC4150937.
5. van Eijk AM, Ayisi JG, Slutsker L, Ter Kuile FO, Rosen DH, Otieno JA, et al. Effect of haematinic supplementation and malaria prevention on maternal anaemia and malaria in western Kenya. Trop Med Int Health. 2007;12(3):342-52. doi: 10.1111/j.1365-3156.2006.01787.x. PubMed PMID: 17313505.
6. Milman N, Jønsson L, Dyre P, Pedersen PL, Larsen LG. Ferrous bisglycinate 25 mg iron is as effective as ferrous sulfate 50 mg iron in the prophylaxis of iron deficiency and anemia during pregnancy in a randomized trial. J Perinat Med. 2014;42(2):197-206. doi: 10.1515/jpm-2013-0153. PubMed PMID: 24152889.
7. Rumbold AR, Crowther CA, Haslam RR, Dekker GA, Robinson JS, Group AS. Vitamins C and E and the risks of preeclampsia and perinatal complications. N Engl J Med. 2006;354(17):1796-806. doi: 10.1056/NEJMoa054186. PubMed PMID: 16641396.
8. Spinnato JA, Freire S, Pinto E Silva JL, Cunha Rudge MV, Martins-Costa S, Koch MA, et al. Antioxidant therapy to prevent preeclampsia: a randomized controlled trial. Obstet Gynecol. 2007;110(6):1311-8. doi: 10.1097/01.AOG.0000289576.43441.1f. PubMed PMID: 18055726.
9. Maged AM, Torky H, Fouad MA, GadAllah SH, Waked NM, Gayed AS, et al. Role of antioxidants in gestational diabetes mellitus and relation to fetal outcome: a randomized controlled trial. J Matern Fetal Neonatal Med. 2016;29(24):4049-54. Epub 20160321. doi: 10.3109/14767058.2016.1154526. PubMed PMID: 26999688.
10. Etheredge AJ, Premji Z, Gunaratna NS, Abioye AI, Aboud S, Duggan C, et al. Iron Supplementation in Iron-Replete and Nonanemic Pregnant Women in Tanzania: A Randomized Clinical Trial. JAMA Pediatr. 2015;169(10):947-55. doi: 10.1001/jamapediatrics.2015.1480. PubMed PMID: 26280534; PubMed Central PMCID: PMC4904713.
11. Jobarteh ML, McArdle HJ, Holtrop G, Sise EA, Prentice AM, Moore SE. mRNA Levels of Placental Iron and Zinc Transporter Genes Are Upregulated in Gambian Women with Low Iron and Zinc Status. J Nutr. 2017;147(7):1401-9. Epub 20170517. doi: 10.3945/jn.116.244780. PubMed PMID: 28515164; PubMed Central PMCID: PMC5483961.

12. Calabuig-Navarro V, Puchowicz M, Glazebrook P, Haghiac M, Minium J, Catalano P, et al. Effect of ω-3 supplementation on placental lipid metabolism in overweight and obese women. Am J Clin Nutr. 2016;103(4):1064-72. doi: 10.3945/ajcn.115.124651. PubMed PMID: 26961929; PubMed Central PMCID: PMC4807706.
13. Villar J, Abdel-Aleem H, Merialdi M, Mathai M, Ali MM, Zavaleta N, et al. World Health Organization randomized trial of calcium supplementation among low calcium intake pregnant women. Am J Obstet Gynecol. 2006;194(3):639-49. doi: 10.1016/j.ajog.2006.01.068. PubMed PMID: 16522392.
14. Vadillo-Ortega F, Perichart-Perera O, Espino S, Avila-Vergara MA, Ibarra I, Ahued R, et al. Effect of supplementation during pregnancy with L-arginine and antioxidant vitamins in medical food on pre-eclampsia in high risk population: randomised controlled trial. BMJ. 2011;342:d2901. Epub 20110519. doi: 10.1136/bmj.d2901. PubMed PMID: 21596735; PubMed Central PMCID: PMC3100912.
15. de Araújo CAL, Ray JG, Figueiroa JN, Alves JG. BRAzil magnesium (BRAMAG) trial: a double-masked randomized clinical trial of oral magnesium supplementation in pregnancy. BMC Pregnancy Childbirth. 2020;20(1):234. Epub 20200421. doi: 10.1186/s12884-020-02935-7. PubMed PMID: 32316938; PubMed Central PMCID: PMC7175576.
16. Castillo-Castrejon M, Yang IV, Davidson EJ, Borengasser SJ, Jambal P, Westcott J, et al. Preconceptional Lipid-Based Nutrient Supplementation in 2 Low-Resource Countries Results in Distinctly Different IGF-1/mTOR Placental Responses. J Nutr. 2021;151(3):556-69. doi: 10.1093/jn/nxaa354. PubMed PMID: 33382407; PubMed Central PMCID: PMC7948206.
17. Hurtado JA, Iznaola C, Peña M, Ruíz J, Peña-Quintana L, Kajarabille N, et al. Effects of Maternal Ω-3 Supplementation on Fatty Acids and on Visual and Cognitive Development. J Pediatr Gastroenterol Nutr. 2015;61(4):472-80. doi: 10.1097/MPG.0000000000000864. PubMed PMID: 25988553.
18. Rytlewski K, Olszanecki R, Lauterbach R, Grzyb A, Basta A. Effects of oral L-arginine on the foetal condition and neonatal outcome in preeclampsia: a preliminary report. Basic Clin Pharmacol Toxicol. 2006;99(2):146-52. doi: 10.1111/j.1742-7843.2006.pto_468.x. PubMed PMID: 16918716.
19. Chiossi G, Quigley MR, Esaka EJ, Novic K, Celebrezze JU, Golde SH, et al. Nutritional supplementation in monochorionic diamniotic twin pregnancies: impact on twin-twin transfusion syndrome. Am J Perinatol. 2008;25(10):667-72. Epub 20081021. doi: 10.1055/s-0028-1091400. PubMed PMID: 18942043.
20. Abramovici A, Gandley RE, Clifton RG, Leveno KJ, Myatt L, Wapner RJ, et al. Prenatal vitamin C and E supplementation in smokers is associated with reduced placental abruption and preterm birth: a secondary analysis. BJOG. 2015;122(13):1740-7. Epub 20141217. doi: 10.1111/1471-0528.13201. PubMed PMID: 25516497; PubMed Central PMCID: PMC4470874.
21. Zheng L, Huang J, Kong H, Wang F, Su Y, Xin H. The effect of folic acid throughout pregnancy among pregnant women at high risk of pre-eclampsia: A randomized clinical trial. Pregnancy Hypertens. 2020;19:253-8. Epub 20200114. doi: 10.1016/j.preghy.2020.01.005. PubMed PMID: 31987769.
22. Jiang X, Bar HY, Yan J, Jones S, Brannon PM, West AA, et al. A higher maternal choline intake among third-trimester pregnant women lowers placental and circulating concentrations of the antiangiogenic factor fms-like tyrosine kinase-1 (sFLT1). FASEB J. 2013;27(3):1245-53. Epub 20121129. doi: 10.1096/fj.12-221648. PubMed PMID: 23195033.

23. Haghiac M, Yang XH, Presley L, Smith S, Dettelback S, Minium J, et al. Dietary Omega-3 Fatty Acid Supplementation Reduces Inflammation in Obese Pregnant Women: A Randomized Double-Blind Controlled Clinical Trial. PLoS One. 2015;10(9):e0137309. Epub 20150904. doi: 10.1371/journal.pone.0137309. PubMed PMID: 26340264; PubMed Central PMCID: PMC4560373.
24. Devi S, Mukhopadhyay A, Dwarkanath P, Thomas T, Crasta J, Thomas A, et al. Combined Vitamin B-12 and Balanced Protein-Energy Supplementation Affect Homocysteine Remethylation in the Methionine Cycle in Pregnant South Indian Women of Low Vitamin B-12 Status. J Nutr. 2017;147(6):1094-103. Epub 20170426. doi: 10.3945/jn.116.241042. PubMed PMID: 28446631.
25. Nakano S, Noguchi T, Takekoshi H, Suzuki G, Nakano M. Maternal-fetal distribution and transfer of dioxins in pregnant women in Japan, and attempts to reduce maternal transfer with Chlorella (Chlorella pyrenoidosa) supplements. Chemosphere. 2005;61(9):1244-55. Epub 20050627. doi: 10.1016/j.chemosphere.2005.03.080. PubMed PMID: 15985279.

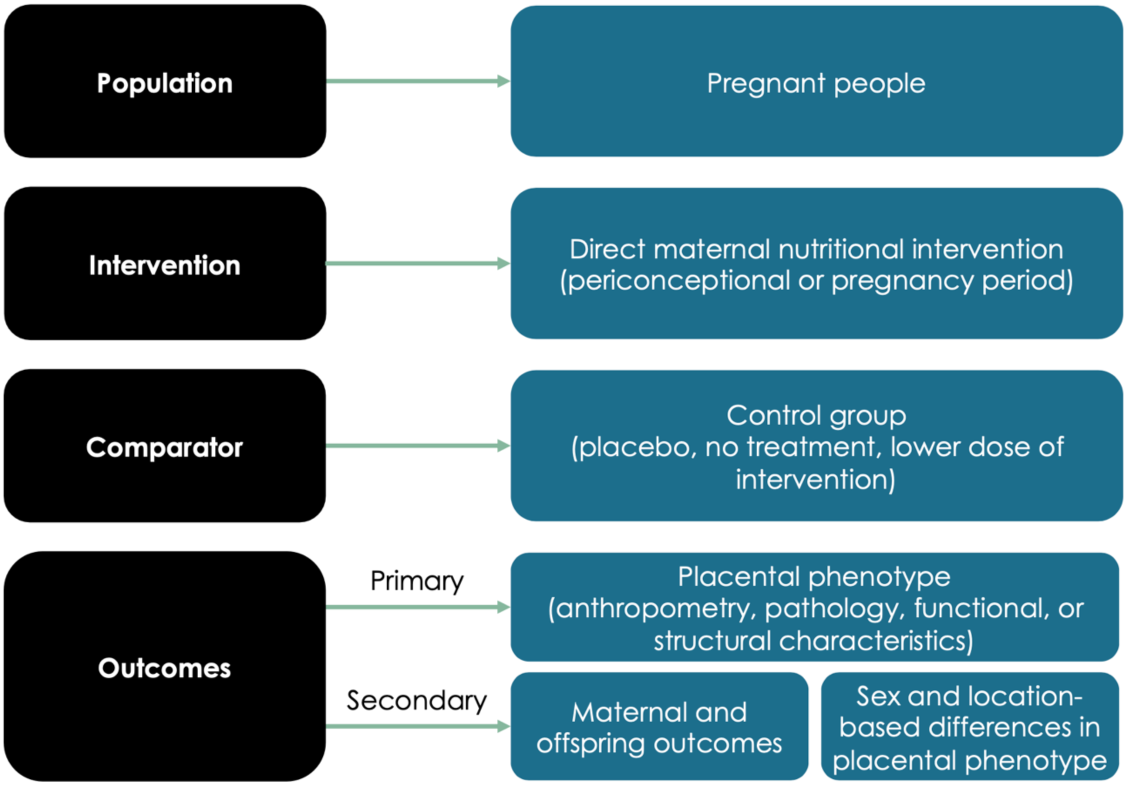

Supplementary Figure S1. Population, intervention, comparator, & outcomes (PICO) criteria for systematic review search.

Supplementary Table S1. Synthesis without meta-analysis (SWiM) framework reporting item checklist.

SWiM reporting item	Item description	Page in manuscript where item is reported
**1** Grouping studies for synthesis	1a) Provide a description of, and rationale for, the groups used in the synthesis (e.g., groupings of populations, interventions, outcomes, study design)	4
	1b) Detail and provide rationale for any changes made subsequent to the protocol in the groups used in the synthesis	5
**2** Describe the standardised metric and transformation methods used	Describe the standardised metric for each outcome. Explain why the metric(s) was chosen, and describe any methods used to transform the intervention effects, as reported in the study, to the standardised metric, citing any methodological guidance consulted	N/A – not possible for the varied types and scope of outcomes reported.
**3** Describe the synthesis methods	Describe and justify the methods used to synthesise the effects for each outcome when it was not possible to undertake a meta-analysis of effect estimates	4
**4** Criteria used to prioritise results for summary and synthesis	Where applicable, provide the criteria used, with supporting justification, to select the particular studies, or a particular study, for the main synthesis or to draw conclusions from the synthesis (e.g., based on study design, risk of bias assessments, directness in relation to the review question)	5-6
**5** Investigation of heterogeneity in reported effects	State the method(s) used to examine heterogeneity in reported effects when it was not possible to undertake a meta-analysis of effect estimates and its extensions to investigate heterogeneity	N/A – studies reported on unique interventions and outcomes
**6** Certainty of evidence	Describe the methods used to assess certainty of the synthesis findings	5-7 – Risk of bias assessment was performed, though synthesis for specific intervention types & outcomes was not possible, given the vast assortment and heterogeneity of outcomes reported
**7** Data presentation methods	Describe the graphical and tabular methods used to present the effects (e.g., tables, forest plots, harvest plots).	6

	Specify key study characteristics (e.g., study design, risk of bias) used to order the studies, in the text and any tables or graphs, clearly referencing the studies included	
**8** Reporting results	For each comparison and outcome, provide a description of the synthesised findings, and the certainty of the findings. Describe the result in language that is consistent with the question the synthesis addresses, and indicate which studies contribute to the synthesis	6-13 – certainty of findings was assessed according to risk of bias assessment
Discussion		
**9** Limitations of the synthesis	Report the limitations of the synthesis methods used and/or the groupings used in the synthesis, and how these affect the conclusions that can be drawn in relation to the original review question	14-15

Supplementary Table S2. Categories of placental measures for the purpose of this review.

Placental measure	Definition	Examples (non-exhaustive)
Anthropometry	Any physical characteristics related to the placenta	Weight, cerebro-placental ratio, fetal-placental ratio, area
Molecular	Any alterations to placental genotype, gene expression, or molecular changes	Placental docosahexaenoic acid content, fatty acid synthase expression
Pathology	Any pathological findings related to the placenta performed by a pathologist	Chorioamnionitis, microscopic placental malaria risk
Placental abruption	Cases of placental abruption	Placental abruption
Placenta-related disease	Any disease with placental origins, or that significantly impacts the placenta	Preeclampsia, premature rupture of membranes, twin-to-twin transfusion syndrome, placental malaria

Supplementary Table S3. Cochrane Collaboration's Tool for Assessing Risk of Bias checklist of assigned criterion.

Checklist item requiring specification	Assigned criterion
Random sequence generation	1. Was the randomization sequence or method reported?
Allocation concealment	1. Was the allocation sequence and participant assignment concealed from parties directly involved (e.g. offsite data manager controlled the randomization assignment)?
Blinding of participants and personnel	1. Were both participants and personnel blinded to which treatment was being received?
Blinding of outcome assessment	1. Was the outcome objective (lower risk of bias) or subjective (higher risk of bias)?
Incomplete outcome data	1. Was placental data provided for each member of the cohort? Or just a selected group?<br>2. If the entire cohort, were attrition rates and acceptability low or high?<br>3. If a subset of the group was selected, how was this subset created?
Selective outcome reporting	1. Are both significant and non-significant results reported?<br>2. Do initial plans for placental data reporting match what was actually reported?
Other (uptake of interventions/intervention adherence)	1. Was uptake and adherence measured and reported by authors?<br>2. How often did researchers follow-up with participants?

Supplementary Table S4. Maternal comorbidities selected for or noted in included studies.

**Maternal comorbidity***	**Number of studies**
None	20
Preeclampsia	12
Obesity/Overweight	7
Hypertension	7
Diabetes	6
Smoking	6
Anemia	3
Nutritional deficiency	3
HIV	2
Allergy status	1

*Some studies selected or noted more than one maternal comorbidity.

Supplementary Table S5. Summary of studies with primary outcomes relevant to maternal health.

First author, year (citation)	Key findings related to maternal health	Significantly altered maternal outcome?*	Significant placental phenotype alterations?*
He, 2020 (1)	No difference to chorioamnionitis rate	No	Yes
de Araújo, 2020 (2)	No difference in preeclampsia or eclampsia, gestational hypertension, maternal stroke, death. Decreased risk of placental abruption (defined as a maternal composite in this study).	Yes	Yes
Zheng, 2020 (3)	Lower rates of preeclampsia in high dose group	Yes	Yes
Hofmeyr, 2019 (4)	No difference in preeclampsia risk	No	No
Ormesher, 2018 (5)	No significant reduction in blood pressure compared to placebo	No	No
Wen, 2018 (6)	No significant differences in preeclampsia risk	No	No
Lager, 2017 (7)	No significant difference to maternal inflammatory status, insulin sensitivity, or circulating lipids	No	Yes
Bujold, 2017 (8)	No significant difference in uterine artery pulse index compared to low-dose group	No	No
Jobarteh, 2017 (9)	Increased maternal iron and zinc status	Yes	Yes
Devi, 2017 (10)	Increased homocysteine remethylation in late pregnancy	Yes	No
Maged, 2016 (11)	Supplementation significantly improved oxidative stress markers (GSH, MDA, SOD, CAT, GPx), improved maternal blood sugar control	Yes	Yes
Calabuig-Navarro, 2016 (12)	Improved maternal inflammatory status; increased maternal plasma n3:n6 ratio, increased maternal plasma enrichment of DHA and EPA	Yes	Yes
Abramovici, 2015 (13)	No difference in preeclampsia risk	No	Yes
Etheredge, 2015 (14)	Significantly improved hemoglobin and iron status. Anemia risk reduced by 40%, risk of iron deficient anemia by 66%.	Yes	No
Hurtado, 2015 (15)	Increased DHA levels and nervonic acid ratio.	Yes	Yes
Haghiac, 2015 (16)	Improved maternal inflammatory status via increased plasma DHA and EPA, and n-3 FA:n-6 FA ratio higher in treatment group.	Yes	Yes
Keelan, 2015 (17)	Increased maternal circulating DHA and EPA levels	Yes	Yes
Kiondo, 2014 (18)	No significant differences in preeclampsia risk	No	No

Milman, 2014 (19)	No significant difference in iron deficiency or iron deficiency anemia between treatment groups	No	No
Parrish, 2013 (20)	No significant differences in preeclampsia risk	No	No
Johnston, 2013 (21)	No significant differences in preeclampsia risk	No	No
Jiang, 2013 (22)	Decreased placental sFLT1 production, which is an antiangiogenic factor linked with preeclampsia	Yes	Yes
Zhou, 2012 (23)	No significant differences in preeclampsia or gestational diabetes mellitus risk	No	No
Vadillo-Ortega, 2011 (24)	Decreased risk of preeclampsia	Yes	Yes
McCance, 2010 (25)	No significant differences in preeclampsia risk	No	No
Roberts, 2010 (26)	No significant difference pregnancy-associated hypertension, thrombocytopenia, elevated serum creatinine levels, or eclamptic seizure	No	No
Villar, 2009 (27)	No significant differences in preeclampsia risk	No	No
Spinnato, 2007 (28)	Increased risk of premature rupture of membranes (PROM), no significant differences in preeclampsia risk	Yes**	Yes
Villar, 2006 (29)	Decreased severity of preeclampsia by 35 weeks' gestation, eclampsia, and hypertension	Yes	Yes
Rumbold, 2006 (30)	No significant differences in preeclampsia risk	No	No

*Significance was determined by the statistical tests performed in each study.
**Alterations to maternal outcomes were adverse effects.

Supplementary Table S6. Summary of studies with primary outcomes relevant to offspring health.

First author, year (citation)	Key findings related to offspring health	Significantly altered offspring outcome?*	Significant placental phenotype alterations?*
Castillo-Castrejon, 2021 (31)	Improved fetal growth due to activated mTOR and IGF-1 signalling in Pakistan cohort	Yes	Yes
He, 2020 (1)	No difference in childhood asthma rates	No	Yes
de Araújo, 2020 (2)	No difference in rates of PT birth, stillbirth, neonatal death, NICU admission, or low birthweight	No	Yes
Brabin, 2019 (32)	Increased preterm birth risk in treatment arm, no difference in growth restriction	Yes**	Yes
Kashanian, 2018 (33)	No difference in preterm rupture of membrane (PROM) or premature preterm rupture of membrane (PPROM) risk	No	No
Maged, 2016 (11)	Decreased fetal MDA, increased SOD. Neonatal birth weight significantly lower in treatment group (3.267kg vs 3.98kg)	Yes	Yes
Abramovici, 2015 (13)	Decreased risk of preterm birth in smokers group	Yes	Yes
Gernand, 2015 (34)	No difference in intrauterine growth factors or birth weight	No	No
Etheredge, 2015 (14)	No difference in birth weight	No	No
Hurtado, 2015 (15)	Increased nervonic acid ratio in plasma and erythrocyte lipids. No difference in visual, cognitive, or psychomotor development.	Yes	Yes
Wietrak, 2015 (35)	No difference to gestational length, birth weight, or Apgar scores	No	Yes
Roberts, 2010 (26)	No difference in preterm birth, fetal-growth restriction, or perinatal death risk	No	No
Winer, 2009 (36)	No difference in birth weight	No	No
Villar, 2009 (27)	No difference in low birth weight, small for gestational age, or perinatal death risk	No	Yes
Chiossi, 2008 (37)	Decreased incidence of twin-to-twin transfusion syndrome (TTTS) and prevalence of TTTS at delivery. Increased time between TTTS diagnosis and delivery.	Yes	Yes
Rumbold, 2006 (30)	No difference in fetal death or small for gestational age risk.	No	No
Rytlewski, 2006 (38)	Decreased risk of intrauterine growth restriction (IUGR). No difference in fetal death risk, birth weight, or gestational length.	Yes	Yes

Villar, 2006 (29)	No difference in preterm birth risk	No	No
Nakano, 2005 (39)	Decreased maternal total toxic equivalents (TEQ), meaning decreased maternal dioxin transfer to child	Yes	No
Helland, 2001 (40)	No difference in gestational length or birth weight	No	No

*Significance was determined by the statistical tests performed in each study.
**Alterations to infant/fetal outcomes were adverse effects.

Supplementary Table S7. Summaries of studies with relevant key placental findings.

First author, year (citation)	Location	Number of groups (group types); n	Treatment intervention	Composition (daily, unless noted otherwise)	Key placental findings following treatment	Significant placental phenotype alterations?*
Castillo-Castrejon, 2021 (31)	Guatemala and Pakistan	4 (1 control and treatment per location); n=12,12,12,12	SQLNS micronutrient supplement	linoleic 4.9 g and α-linolenic 0.59 g	-Average placental area (cm^2) larger in treatment groups<br>-In Pakistani cohort, increased rpS6(T37/46):rpS6 (ribosomal protein S6) ratio 1.5 fold, decreased AMPKA(T172):AMPKA (AMP-activated protein kinase) ratio<br>-Increased pregnancy-associated plasma protein A (PAPP-A) in both treatment cohorts	Yes
Awe, 2020 (41)	United States of America (USA)	1, split into D3 sufficient and deficient after treatment (treatment); n=43	Vitamin D3 + standard prenatal vitamin	4000IU D3 + 400IU from prenatal	-Near delivery, lower placental Fms Related Receptor Tyrosine Kinase 1 (Flt-1), SRC associated in mitosis of 68kDA (Sam68) than Vitamin D3 deficient placentas<br>-No difference in sFLT-1	Yes
He, 2020 (1)	USA	2 (<32 ng/mL serum hydro cholecalciferol status, >32 ng/mL); n=27,20	Vitamin D	400 vs 4000 IU Vitamin D	-No difference in placental abruption risk<br>- Lower Integrator Complex Subunit 9 (INTS9), von Willebrand factor (vWF), Metastasis Associated in Colon Cancer-1 (MACC1), Age-related Maculopathy Susceptibility 2 (ARMS2) expression<br>-Increased contactin 5 (CNTN5) gene expression, chorionic villi surface density in higher concentration group	Yes
de Araújo, 2020 (2)	Brazil	2 (placebo, treatment); n=422,407	Magnesium citrate	300 mg magnesium citrate	-Lower placental abruption risk (2.2% vs. 5.0%)	Yes

Zheng, 2020 (3)	China	2 (low dose, high dose); n=378,410	Folic Acid	0.4mg FA, 4mg FA	-Lower preeclampsia risk with high treatment compliance<br>-No difference in placental abruption risk	Yes
Brabin, 2019 (32)	Burkina Faso	2 (control, treatment); n=144,163	Iron + folic acid	60mg ferrous gluconate + 2.8mg folic acid	-Reduced grade 2 and 3 chorioamnionitis risk (40.4% vs 48.9%)<br>-No difference in placental malaria risk	Yes
Hofmeyr, 2019 (4)	South Africa, Zimbabwe, Argentina	2 (placebo, treatment); n=283,298	Calcium	500 mg calcium	-No difference in placental abruption risk	No
Ormesher, 2018 (5)	United Kingdom (UK)	2 (placebo, treatment); n=21,20	Beetroot juice	~400mg nitrate in 70mL beetroot juice	-No difference in uteroplacental blood flow	No
Wen, 2018 (6)	Argentina, Australia, Canada, Jamaica, UK	2 (placebo, treatment); n=1179,1172	Folic acid	4.0mg from 8-16 weeks, 1.1mg until birth	-No difference in placental abruption risk	No
Kashanian, 2018 (33)	Iran	2 (placebo, treatment); n=111, 127	Copper	1000mg copper	-No difference in placental abruption or placenta previa risk	No
Lager, 2017 (7)	USA	2 (placebo, treatment); n=19,19	DHA	DHA 800mg w/ algal oil, placebo was corn/soy oil.	-Increased placental membrane DHA levels, fatty acid transporting protein 4 expression<br>-Decreased placental inflammation via decreased nuclear factor of kappa light polypeptide gene enhancer in B-cells inhibitor alpha (IkBa), p38 mitogen-activated protein kinase alpha (p38α), and increased cyclo-oxygenase 2 (COX-2) and nuclear factor kappa B p65 activation (NF-kB)<br>-Decreased fetal-placental ratio<br>-No difference in placental weight	Yes

Park, 2017 (42)	USA	1 (treatment); n=24	Vitamin D	511IU (311IU from diet, 200IU via multivitamin)	-Increased LDL Receptor Related Protein 2 (LRP2), cubulin (CUBN), Cytochrome P450 Family 2 Subfamily R Member 1 (CYP2R1), Cytochrome P450 Family 24 Subfamily A Member 1 (CYP24A1), Cytochrome P450 Family 27 Subfamily B Member 1 abundance (CYP27B1)	Yes
Bujold, 2017 (8)	Canada	2 (low dose, high dose); n=131 total	Low or high dose flavanol and theobromine chocolate	69mg Theobromine, 25.4mg Epicatechins, 6.3mg Catechins, 32.1mg Dimers, 107.6mg Trimers-decamers	-No difference in preeclampsia risk via uterine artery pulsatility index	No
Jobarteh, 2017 (9)	Gambia	4 (iron + folate (FeFol), MMN, PEB, PEB+MMN); n=74,76,76,75	Multi-micronutrient (MMN) and/or Protein-Energy (PEB) Ball	FeFol: 60mg/d, 400 ug folic acid. MMNs: combination of 15 micronutrients. Pe: same as FeFol plus energy, protein, lipids. PE + MMNs: MMNs content plus PE content.	-Placental iron uptake protein transferrin receptor 1 mRNA levels 30-49% higher in PE and PE+MMN arms than FeFol arm. 29% higher in PE+MMN arm than just MMN arm.<br>-No difference in placental weight, breadth, or length	Yes

Devi, 2017 (10)	India	3 (Placebo, placebo+milk, milk+ B12); n=31,29,29	Vitamin B-12	500 mL/d milk, and/or 100ug vitamin b-12 tablet/d	-No difference in placental mRNA for methionine pathways, placental long interspersed nuclear elements 1 (LINE-1), and vascular endothelial growth factor (VEGF) promoter methylation.	No
Darling, 2017 (43)	Tanzania	4 (placebo, VitA, Zinc, VitA+Zinc); n=611,613,608, 602	Vitamin A and/or Zinc	2500 IU VitA, and/or 25mg zinc	-36% lower risk of histopathological-positive placental malaria result with zinc supplementation	Yes
Maged, 2016 (11)	Egypt	2 (control, treatment); n=100,100	Vitamin C	1 gram L-ascorbic acid	-Lower glutathione (GSH), malondialdehyde (MDA), catalase (CAT), glutathione peroxidase (GPx)<br>-Higher superoxide dismutase (SOD)	Yes
Calabuig-Navarro, 2016 (12)	USA	2 (placebo, treatment); n=16,17	LC-PUFA	2000 mg n-3LCPUFA (800mg DHA + 1200mg EPA)	-Lower fatty acid synthase (FAS), diacylglycerol O-acyltransferase 1 (DGAT1), peroxisome proliferator–activated receptor γ (PPAR), perilipin 2 (PLIN2)	Yes
Johnston, 2016 (44)	Ireland	2 (placebo, treatment); n=30,27	Vitamin C + E	1000mg vitamin C, 400IU Vitamin E	-No difference in placental antioxidant enzymes and lipid peroxidation	No
Owens, 2015 (45)	Gambia	2 (placebo, treatment); n=247,239	Multi-micronutrient (UNIMMAP)	Vitamin A (800 retinol equivalents), D (200 IU), E (10 mg), C (70 mg), thiamin (1.4 mg), riboflavin (1.4 mg), niacin (18 mg),	-No difference in plasminogen activator inhibitor-1 (PAI-1), plasminogen activator inhibitor-2 (PAI-2), PAI-1:PAI-2 ratio<br>-No difference in placental weight	No

				pyridoxine (1.9 mg), cobalamin (2.6 mg), folic acid (400 mg), iron (30 mg), zinc (15 mg), copper (2 mg), selenium (65 mg), iodine (150 mg)		
Abramovici, 2015 (13)	USA	4 (smoker placebo, smoker treatment, non-smoker placebo, non-smoker treatment); n=763,788,4213,4205	Vitamin C + E	1000 mg Vitamin C, 400 IU vitamin E	-Reduced placental abruption risk in smoker cohorts (1.5% vs 0.1%)<br>-No difference in preeclampsia risk	Yes
Gernand, 2015 (34)	Bangladesh	2 (Iron + folic acid (IFA), MM); n=191,205	Multi-micronutrient	IFA: 27mg iron + 600ug FA<br>MM: IFA + vitamins A (770 µg retinol equivalents), D (5 µg), E (15 mg), B12(2.5 mg), B6 (1.9 mg), C (85 mg), thiamin (1.4 mg),	-No difference in placental weight	No

				riboflavin (1.4 mg), niacin (1.4 mg), zinc (12 mg), iodine (220 µg), copper (1000 µg), selenium (60 µg)		
Etheredge, 2015 (14)	Tanzania	2 (placebo, treatment); n=510,493	Iron	60 mg iron	-No difference in weight, or microscopic and submicroscopic placental malaria risk	
Hurtado, 2015 (15)	Spain	2 (control, treatment); n=38,38	Fish oil	18 mg/100mL EPA, 80 mg/mL DHA	-Higher placental fatty acid composition in phospholipids of elaidic acid (C18:ln-9), linoleic acid (C18:2n-6), docosahexaenoic acid (DHA), omega-3 polyunsaturated fatty acid (n-3 PUFA)<br>-No difference to alpha-linolenic acid (C18:3n-3), 15-tetracosenoic acid (C24:1n-9), eicosapentaenoic acid (EPA), saturated fatty acid (SFA), monounsaturated fatty acid (MUFA), omega-6 polyunsaturated fatty acid (n-6 PUFA)	Yes
Haghiac, 2015 (16)	USA	2 (placebo, treatment); n=25,24	Omega-3 fatty acid supplement	800mg DHA and 1200mg EPA	-Lower placental interleukin 6 and 8 (IL6, IL8) tumour necrosis factor alpha (TNFα), toll-like receptor 4 (TLR4) mRNA expression<br>-No difference to total omega-3 fatty acid (n-3 PUFA) concentration	Yes
Keelan, 2015 (17)	Australia	2 (placebo, treatment); n=28,22	Omega-3 polyunsaturated fatty acids (n-3 PUFAs)	4 pills, 3.7g n-3 PUFAS (56% DHA, 27.7% EPA) per tablet	-Increased placental DHA (80%), TNFα (14x), 18-hydroxyeicosapentaenoic acid (18-HEPE)	Yes

					-No difference to interleukin 1beta, 6, or 10 (IL1B, IL6, IL10), Prostaglandin-Endoperoxide Synthase 2 (PTGS2)	
Wietrak, 2015 (35)	Poland	2 (control, treatment); n=50,28	DHA	300 mg DHA	-Lower average placental weight (510g vs 530g)<br>-No difference in protein expression of cyclin-dependent kinase inhibitor 1 (p21) or antigen Ki-67	Yes
Kiondo, 2014 (18)	Uganda	2 (placebo, treatment); n=418,415	Vitamin C	1000mg Vitamin C	-No difference in preeclampsia incidence or placental abruption risk	No
Milman, 2014 (19)	New Zealand	2 (ferrous bisglycinate, ferrous sulfate); n=40,40	Iron	25mg ferrous bisglycinate elemental iron, or 50mg ferrous sulfate elemental iron	-No difference in immunostaining for transferrin receptor	No
Parrish, 2013 (20)	USA	2 (placebo, treatment); n=135,132	Phytonutrient	~7.5mg beta-carotene, 234mg vitamin C, 30mg vitamin E, 420mg folate, 60mg calcium	-No difference in placental abruption or PPROM risk	No
Johnston, 2013 (21)	Ireland	2 (placebo, treatment); n=382,379	Vitamin C + E	1000mg Vitamin C, 400 IU vitamin E	-No difference in placental abruption risk	No
Jiang, 2013 (22)	USA	2 (low dose, high dose); n=12,12	Choline	480mg/d or 930mg/d choline through	-30% downregulation of soluble fms-like tyrosine kinase-1 (sFLT1)<br>-43 placental genes upregulated, of note ghrelin and obestatin prepropeptide	Yes

				380mg/d from diet, plus either 100 or 550mg from supplemental choline chloride.	(GHRL; 1.6x increase), neuropeptide Y (NPY) receptor 75 (1.7x increase), elastin (2.03x increase)<br>-123 genes downregulated<br>-Treatment alters 197 biological processes in higher dose	
Jiang, 2012 (46)	USA	2 (low dose, high dose); n=12,12	Choline	480mg/d or 930mg/d choline through 380mg/d from diet, plus either 100 or 550mg from supplemental choline chloride.	-Increased placental promoter methylation of cortisol-regulating genes, corticotropin releasing hormone (CRH) and glucocorticoid receptor (NR3C1)<br>-Increased placental global DNA methylation and demethylated histone H3 at H3K9me2<br>-Lower placental corticotropin-releasing hormone (CRH) transcript abundance	Yes
Zhou, 2012 (23)	Australia	2 (control, treatment); n=1202,1197	DHA fish oil	3 pills, 500mg each totalling to 800mg DHA	-No difference in placental weight or preeclampsia risk	No
Vadillo-Ortega, 2011 (24)	Mexico	3 (placebo, L-arg+vitamins, vitamins); n=222,228,222	L-arginine (L-arg) and/or vitamins	L-arg bar: 6.6 g L-arg, plus vitamins from vitamin-only bar (listed next) from 2 bars. Vitamins: no L-arg but other vitamins: C	-Lower preeclampsia risk (30% in placebo vs 13% in L-arg + vitamin group vs 23% in vitamin group)<br>-No difference in placental abruption risk	Yes

				(250mg), B6 (2.0mg), B12 (4.8ug), E (200IU), niacin (25mg), folate (200ug).		
McCance, 2010 (25)	Ireland, Scotland, England	2 (placebo, treatment); n=382,379	Vitamin C + E	1000mg Vitamin C, 400 IU vitamin E	-No difference in placental abruption or preeclampsia risk	No
Mercer, 2010 (47)	USA	2 (placebo, treatment); n=39,34	Vitamin C + E	500mg Vitamin C, 200IU vitamin E	-No difference in amnio-choriodecidua separation risk	No
Roberts, 2010 (26)	USA	2 (placebo, treatment); n=4976,4993	Vitamin C + E	1000mg Vitamin C, 400IU Vitamin E	-No difference in placental abruption or preeclampsia risk	No
Winer, 2009 (36)	France	2 (placebo, treatment); n=22,21	L-arginine	14g (90cc) ARG	-No difference in placental weight, placental abruption, hypotrophy, thrombosis, or histological abnormality risk	No
Villar, 2009 (27)	India, Peru, South Africa, Vietnam	2 (placebo, treatment); n=678,687	Vitamin C + E	1000mg Vitamin C, 400 IU vitamin E	-No difference in placental abruption or preeclampsia risk	No
Chiossi, 2008 (37)	USA	2 (standard diet, treatment); n=51,52	Ensure liquid nutritional supplement	250kcal, 6g lipids, 40g carbohydrates, 9g protein, vitamins, minerals	-Decreased risk of Twin-to-twin transfusion syndrome (TTTS) diagnosis (15.5% vs 38%), and TTTS at delivery (11.8% vs 34.6%)	Yes

Spinnato, 2007 (28)	Brazil	2 (placebo, treatment); n=349,351	Vitamin C + E	1000mg Vitamin C, 400IU vitamin E	-Increased risk of PROM (5.5% vs 10.6%)<br>-No difference in placental abruption risk	Yes
Kaplas, 2007 (48)	Finland	3 (control/placebo, diet/placebo, diet/probiotics); n=8,12,10	Diet and/or probiotics	Diet: Overall monounsaturated fatty acids (MUFA) contributing 10–15%, PUFA 5–10%, and saturated fatty acids (SFA) 10% or less of energy in-take. Probiotics: capsules containing Lactobacillus rhamnosus GG and Bifidobacterium lactis Bb12. Exact composition not listed.	-Increased n-3 PUFA, Dihomo-c-linolenic acid (DHGA), and eicosatetraenoic acid (20:4n-3) in diet group<br><br>-Increased DHGA, EPA, arachidonic acid, linoleic acid (LA), 20:4n-3 in probiotic group. Changes to DHGA and linoleic acid attributed to probiotics only.	Yes

van Eijk, 2007 (49)	Kenya	3 groups each separated into 2 for HIV+ or HIV- status (0 intervention, 1, 2); n=1172,1140,796	Haematinics and/or IPTp-SP	Haematinics: 200 mg ferrous sulphate + 5mg folic acid. IPTp-SP: intermittent preventative treatment with sulphadoxine-pyrimethamine	-Decreased placental malaria risk for group using 2 interventions compared to reference group of no intervention (0.56 overall, 0.43 for HIV+, 0.61 for HIV-)	Yes
Larqué, 2006 (50)	Spain	4 (placebo, methyltetrahydro-folate (MTHF), DHA, DHA+MTHF); n=26,27,25,21	MTHF and/or DHA	MTHF: 400ug 5-methyl-tetrahydrofolic acid, DHA: 500mg DHA + 150mg eicosapentaenoic acid	-Increased DHA in placental phospholipids at delivery, proportion of DHA in placental phospholipids and mRNA expression in the membrane proteins fatty acid transport proteins 1 and 4 (FATP-1, FATP-4)<br>-No difference in placental tissue mRNA expression of FATP-1, FATP-4, FATP-6, fatty acid translocase (FAT/CD36), plasma membrane fatty acid–binding protein (FABPpm/GOT2), heart fatty-acid transport protein (H-FABP), and arachidonic acid (AA) proportion<br>-No difference in placental weight	Yes
Rytlewski, 2006 (38)	Poland	2 (placebo, treatment); n=31,30	L-arginine	3g L-arginine (6*0.5g tablets)	-Increased cerebro-placental ratio after 4 weeks (1.5 to 1.71 vs 1.41 to 1.09)	Yes

Klingler, 2006 (51)	Spain	4 (placebo, fish oil+5-MTHF, fish oil, 5-MTHF); n=12,11,16,16	Modified fish oil and/or MTHF	Fish oil: 500mg DHA + 150mg EPA. MTHF: 400ug 5-MTHF (delivered as 800ug 6,RS,5-methyltetrahydrofolate)	-Proportion of DHA in placental phospholipids similar between fish oil groups, but higher than non-fish oil groups<br>-Increased proliferation cell nuclear antigen (PCNA) in placentas of fish oil + 5-MTHF group than placebo by 66%<br>-Increased EPA in fish oil groups<br>-No difference in placental p53 or cytokeratin levels	Yes
Rumbold, 2006 (30)	Australia	2 (placebo, treatment); n=942,935	Vitamin C + E	1000mg Vitamin C + 400 IU Vitamin E (from a combined 4 pills)	-No difference in placental abruption or preeclampsia risk	No
Villar, 2006 (29)	Argentina, Egypt, South Africa	2 (placebo, treatment); n=4161,4151	Calcium	1.5g calcium	-Decreased risk of preeclampsia by 35 weeks gestation (1.2% vs 2.8%)<br>-No difference in placental abruption risk	Yes
Cox, 2005 (52)	Ghana	2 (placebo, treatment); n=38,38	Vitamin A	10,000IU vitamin A as retinyl palmitate	-No difference in active or chronic-active placental malaria risk in current pregnancy compared to past resolved infection at delivery	No
Nakano, 2005 (39)	Japan	2 (control, treatment); n=21, 23	Chlorella	6g chlorella (30 tablets/day, 10 after each meal), composed of (in g/100g): moisture, 4.7;	-No difference in pg/whole sample g for polychlorinated dibenzo-p-dioxins (PCDD), poly-chlorinated dibenzofurans (PCDF), co-planar polychlorinated biphenyls (Co-PCB), total toxic equivalents (TEQ)	No

				chlorophyll, 2.4; dietary fiber, 9.8; protein (N · 6.5), 57.8; lipid, 10.4.		
Pressman, 2003 (53)	USA	2 (control, treatment); n=10,10	Prenatal vitamin plus Vitamin C + E	Prenatal (120mg Vitamin C + 30 IU vitamin E) + 400 IU vitamin E + 500 mg vitamin C	-No difference in chorioamnion grams to burst (gf), chorioamnion maximal deflection (mm), or chorioamnion Vitamin E levels	No
Helland, 2001 (40)	Norway	2 (placebo, treatment); n=166,175	Cod liver oil	10mL cod oil (117ug/mL vitamin A, 1 ug/mL Vitamin D, 1.4 mg/mL dl-alpha-tocopherol	-No difference in placental weight	No

*Significance was determined by the statistical tests performed in each study.

References

1. He M, Mirzakhani H, Chen L, Wu R, Litonjua AA, Bacharier L, et al. Vitamin D Sufficiency Has a Limited Effect on Placental Structure and Pathology: Placental Phenotypes in the VDAART Trial. Endocrinology. 2020;161(6). doi: 10.1210/endocr/bqaa057. PubMed PMID: 32270179; PubMed Central PMCID: PMC7528633.
2. de Araújo CAL, Ray JG, Figueiroa JN, Alves JG. BRAzil magnesium (BRAMAG) trial: a double-masked randomized clinical trial of oral magnesium supplementation in pregnancy. BMC Pregnancy Childbirth. 2020;20(1):234. Epub 20200421. doi: 10.1186/s12884-020-02935-7. PubMed PMID: 32316938; PubMed Central PMCID: PMC7175576.
3. Zheng L, Huang J, Kong H, Wang F, Su Y, Xin H. The effect of folic acid throughout pregnancy among pregnant women at high risk of pre-eclampsia: A randomized clinical trial. Pregnancy Hypertens. 2020;19:253-8. Epub 20200114. doi: 10.1016/j.preghy.2020.01.005. PubMed PMID: 31987769.
4. Hofmeyr GJ, Betrán AP, Singata-Madliki M, Cormick G, Munjanja SP, Fawcus S, et al. Prepregnancy and early pregnancy calcium supplementation among women at high risk of pre-eclampsia: a multicentre, double-blind, randomised, placebo-controlled trial. Lancet. 2019;393(10169):330-9. doi: 10.1016/s0140-6736(18)31818-x. PubMed PMID: 30696573; PubMed Central PMCID: PMC6346082.
5. Ormesher L, Myers JE, Chmiel C, Wareing M, Greenwood SL, Tropea T, et al. Effects of dietary nitrate supplementation, from beetroot juice, on blood pressure in hypertensive pregnant women: A randomised, double-blind, placebo-controlled feasibility trial. Nitric Oxide. 2018;80:37-44. Epub 20180809. doi: 10.1016/j.niox.2018.08.004. PubMed PMID: 30099096.
6. Wen SW, White RR, Rybak N, Gaudet LM, Robson S, Hague W, et al. Effect of high dose folic acid supplementation in pregnancy on pre-eclampsia (FACT): double blind, phase III, randomised controlled, international, multicentre trial. Bmj. 2018;362:k3478. Epub 20180912. doi: 10.1136/bmj.k3478. PubMed PMID: 30209050; PubMed Central PMCID: PMC6133042.
7. Lager S, Ramirez VI, Acosta O, Meireles C, Miller E, Gaccioli F, et al. Docosahexaenoic Acid Supplementation in Pregnancy Modulates Placental Cellular Signaling and Nutrient Transport Capacity in Obese Women. J Clin Endocrinol Metab. 2017;102(12):4557-67. doi: 10.1210/jc.2017-01384. PubMed PMID: 29053802; PubMed Central PMCID: PMC5718695.
8. Bujold E, Leblanc V, Lavoie-Lebel É, Babar A, Girard M, Poungui L, et al. High-flavanol and high-theobromine versus low-flavanol and low-theobromine chocolate to improve uterine artery pulsatility index: a double blind randomized clinical trial. J Matern Fetal Neonatal Med. 2017;30(17):2062-7. Epub 20161003. doi: 10.1080/14767058.2016.1236250. PubMed PMID: 27696933.
9. Jobarteh ML, McArdle HJ, Holtrop G, Sise EA, Prentice AM, Moore SE. mRNA Levels of Placental Iron and Zinc Transporter Genes Are Upregulated in Gambian Women with Low Iron and Zinc Status. J Nutr. 2017;147(7):1401-9. Epub 20170517. doi: 10.3945/jn.116.244780. PubMed PMID: 28515164; PubMed Central PMCID: PMC5483961.
10. Devi S, Mukhopadhyay A, Dwarkanath P, Thomas T, Crasta J, Thomas A, et al. Combined Vitamin B-12 and Balanced Protein-Energy Supplementation Affect Homocysteine Remethylation in the Methionine Cycle in Pregnant South Indian Women of Low Vitamin B-12 Status. J Nutr. 2017;147(6):1094-103. Epub 20170426. doi: 10.3945/jn.116.241042. PubMed PMID: 28446631.
11. Maged AM, Torky H, Fouad MA, GadAllah SH, Waked NM, Gayed AS, et al. Role of antioxidants in gestational diabetes mellitus and relation to fetal outcome: a randomized controlled

trial. J Matern Fetal Neonatal Med. 2016;29(24):4049-54. Epub 20160321. doi: 10.3109/14767058.2016.1154526. PubMed PMID: 26999688.
12. Calabuig-Navarro V, Puchowicz M, Glazebrook P, Haghiac M, Minium J, Catalano P, et al. Effect of ω-3 supplementation on placental lipid metabolism in overweight and obese women. Am J Clin Nutr. 2016;103(4):1064-72. doi: 10.3945/ajcn.115.124651. PubMed PMID: 26961929; PubMed Central PMCID: PMC4807706.
13. Abramovici A, Gandley RE, Clifton RG, Leveno KJ, Myatt L, Wapner RJ, et al. Prenatal vitamin C and E supplementation in smokers is associated with reduced placental abruption and preterm birth: a secondary analysis. Bjog. 2015;122(13):1740-7. Epub 20141217. doi: 10.1111/1471-0528.13201. PubMed PMID: 25516497; PubMed Central PMCID: PMC4470874.
14. Etheredge AJ, Premji Z, Gunaratna NS, Abioye AI, Aboud S, Duggan C, et al. Iron Supplementation in Iron-Replete and Nonanemic Pregnant Women in Tanzania: A Randomized Clinical Trial. JAMA Pediatr. 2015;169(10):947-55. doi: 10.1001/jamapediatrics.2015.1480. PubMed PMID: 26280534; PubMed Central PMCID: PMC4904713.
15. Hurtado JA, Iznaola C, Peña M, Ruíz J, Peña-Quintana L, Kajarabille N, et al. Effects of Maternal Ω-3 Supplementation on Fatty Acids and on Visual and Cognitive Development. J Pediatr Gastroenterol Nutr. 2015;61(4):472-80. doi: 10.1097/mpg.0000000000000864. PubMed PMID: 25988553.
16. Haghiac M, Yang XH, Presley L, Smith S, Dettelback S, Minium J, et al. Dietary Omega-3 Fatty Acid Supplementation Reduces Inflammation in Obese Pregnant Women: A Randomized Double-Blind Controlled Clinical Trial. PLoS One. 2015;10(9):e0137309. Epub 20150904. doi: 10.1371/journal.pone.0137309. PubMed PMID: 26340264; PubMed Central PMCID: PMC4560373.
17. Keelan JA, Mas E, D'Vaz N, Dunstan JA, Li S, Barden AE, et al. Effects of maternal n-3 fatty acid supplementation on placental cytokines, pro-resolving lipid mediators and their precursors. Reproduction. 2015;149(2):171-8. Epub 20141212. doi: 10.1530/rep-14-0549. PubMed PMID: 25504868.
18. Kiondo P, Wamuyu-Maina G, Wandabwa J, Bimenya GS, Tumwesigye NM, Okong P. The effects of vitamin C supplementation on pre-eclampsia in Mulago Hospital, Kampala, Uganda: a randomized placebo controlled clinical trial. BMC Pregnancy Childbirth. 2014;14:283. Epub 20140821. doi: 10.1186/1471-2393-14-283. PubMed PMID: 25142305; PubMed Central PMCID: PMC4150937.
19. Milman N, Jønsson L, Dyre P, Pedersen PL, Larsen LG. Ferrous bisglycinate 25 mg iron is as effective as ferrous sulfate 50 mg iron in the prophylaxis of iron deficiency and anemia during pregnancy in a randomized trial. J Perinat Med. 2014;42(2):197-206. doi: 10.1515/jpm-2013-0153. PubMed PMID: 24152889.
20. Parrish MR, Martin JN, Jr., Lamarca BB, Ellis B, Parrish SA, Owens MY, et al. Randomized, placebo controlled, double blind trial evaluating early pregnancy phytonutrient supplementation in the prevention of preeclampsia. J Perinatol. 2013;33(8):593-9. Epub 20130228. doi: 10.1038/jp.2013.18. PubMed PMID: 23448939.
21. Johnston PC, Powell LA, McCance DR, Pogue K, McMaster C, Gilchrist S, et al. Placental protein tyrosine nitration and MAPK in type 1 diabetic pre-eclampsia: Impact of antioxidant vitamin supplementation. J Diabetes Complications. 2013;27(4):322-7. Epub 20130402. doi: 10.1016/j.jdiacomp.2013.02.001. PubMed PMID: 23558107.
22. Jiang X, Bar HY, Yan J, Jones S, Brannon PM, West AA, et al. A higher maternal choline intake among third-trimester pregnant women lowers placental and circulating concentrations of

the antiangiogenic factor fms-like tyrosine kinase-1 (sFLT1). Faseb j. 2013;27(3):1245-53. Epub 20121129. doi: 10.1096/fj.12-221648. PubMed PMID: 23195033.
23. Zhou SJ, Yelland L, McPhee AJ, Quinlivan J, Gibson RA, Makrides M. Fish-oil supplementation in pregnancy does not reduce the risk of gestational diabetes or preeclampsia. Am J Clin Nutr. 2012;95(6):1378-84. Epub 20120502. doi: 10.3945/ajcn.111.033217. PubMed PMID: 22552037.
24. Vadillo-Ortega F, Perichart-Perera O, Espino S, Avila-Vergara MA, Ibarra I, Ahued R, et al. Effect of supplementation during pregnancy with L-arginine and antioxidant vitamins in medical food on pre-eclampsia in high risk population: randomised controlled trial. Bmj. 2011;342:d2901. Epub 20110519. doi: 10.1136/bmj.d2901. PubMed PMID: 21596735; PubMed Central PMCID: PMC3100912.
25. McCance DR, Holmes VA, Maresh MJ, Patterson CC, Walker JD, Pearson DW, et al. Vitamins C and E for prevention of pre-eclampsia in women with type 1 diabetes (DAPIT): a randomised placebo-controlled trial. Lancet. 2010;376(9737):259-66. Epub 20100626. doi: 10.1016/S0140-6736(10)60630-7. PubMed PMID: 20580423; PubMed Central PMCID: PMC2911677.
26. Roberts JM, Myatt L, Spong CY, Thom EA, Hauth JC, Leveno KJ, et al. Vitamins C and E to prevent complications of pregnancy-associated hypertension. N Engl J Med. 2010;362(14):1282-91. doi: 10.1056/NEJMoa0908056. PubMed PMID: 20375405; PubMed Central PMCID: PMC3039216.
27. Villar J, Purwar M, Merialdi M, Zavaleta N, Thi Nhu Ngoc N, Anthony J, et al. World Health Organisation multicentre randomised trial of supplementation with vitamins C and E among pregnant women at high risk for pre-eclampsia in populations of low nutritional status from developing countries. Bjog. 2009;116(6):780-8. doi: 10.1111/j.1471-0528.2009.02158.x. PubMed PMID: 19432566.
28. Spinnato JA, 2nd, Freire S, Pinto ESJL, Cunha Rudge MV, Martins-Costa S, Koch MA, et al. Antioxidant therapy to prevent preeclampsia: a randomized controlled trial. Obstet Gynecol. 2007;110(6):1311-8. doi: 10.1097/01.AOG.0000289576.43441.1f. PubMed PMID: 18055726.
29. Villar J, Abdel-Aleem H, Merialdi M, Mathai M, Ali MM, Zavaleta N, et al. World Health Organization randomized trial of calcium supplementation among low calcium intake pregnant women. Am J Obstet Gynecol. 2006;194(3):639-49. doi: 10.1016/j.ajog.2006.01.068. PubMed PMID: 16522392.
30. Rumbold AR, Crowther CA, Haslam RR, Dekker GA, Robinson JS. Vitamins C and E and the risks of preeclampsia and perinatal complications. N Engl J Med. 2006;354(17):1796-806. doi: 10.1056/NEJMoa054186. PubMed PMID: 16641396.
31. Castillo-Castrejon M, Yang IV, Davidson EJ, Borengasser SJ, Jambal P, Westcott J, et al. Preconceptional Lipid-Based Nutrient Supplementation in 2 Low-Resource Countries Results in Distinctly Different IGF-1/mTOR Placental Responses. J Nutr. 2021;151(3):556-69. doi: 10.1093/jn/nxaa354. PubMed PMID: 33382407; PubMed Central PMCID: PMC7948206.
32. Brabin B, Gies S, Roberts SA, Diallo S, Lompo OM, Kazienga A, et al. Excess risk of preterm birth with periconceptional iron supplementation in a malaria endemic area: analysis of secondary data on birth outcomes in a double blind randomized controlled safety trial in Burkina Faso. Malar J. 2019;18(1):161. Epub 20190506. doi: 10.1186/s12936-019-2797-8. PubMed PMID: 31060615; PubMed Central PMCID: PMC6501288.
33. Kashanian M, Hadizadeh H, Faghankhani M, Nazemi M, Sheikhansari N. Evaluating the effects of copper supplement during pregnancy on premature rupture of membranes and pregnancy

outcome. J Matern Fetal Neonatal Med. 2018;31(1):39-46. Epub 20170118. doi: 10.1080/14767058.2016.1274299. PubMed PMID: 28002986.
34. Gernand AD, Schulze KJ, Nanayakkara-Bind A, Arguello M, Shamim AA, Ali H, et al. Effects of Prenatal Multiple Micronutrient Supplementation on Fetal Growth Factors: A Cluster-Randomized, Controlled Trial in Rural Bangladesh. PLoS One. 2015;10(10):e0137269. Epub 20151002. doi: 10.1371/journal.pone.0137269. PubMed PMID: 26431336; PubMed Central PMCID: PMC4591978.
35. Wietrak E, Kamiński K, Leszczyńska-Gorzelak B, Oleszczuk J. Effect of Docosahexaenoic Acid on Apoptosis and Proliferation in the Placenta: Preliminary Report. Biomed Res Int. 2015;2015:482875. Epub 20150803. doi: 10.1155/2015/482875. PubMed PMID: 26339616; PubMed Central PMCID: PMC4538367.
36. Winer N, Branger B, Azria E, Tsatsaris V, Philippe HJ, Rozé JC, et al. L-Arginine treatment for severe vascular fetal intrauterine growth restriction: a randomized double-bind controlled trial. Clin Nutr. 2009;28(3):243-8. Epub 20090408. doi: 10.1016/j.clnu.2009.03.007. PubMed PMID: 19359073.
37. Chiossi G, Quigley MR, Esaka EJ, Novic K, Celebrezze JU, Golde SH, et al. Nutritional supplementation in monochorionic diamniotic twin pregnancies: impact on twin-twin transfusion syndrome. Am J Perinatol. 2008;25(10):667-72. Epub 20081021. doi: 10.1055/s-0028-1091400. PubMed PMID: 18942043.
38. Rytlewski K, Olszanecki R, Lauterbach R, Grzyb A, Basta A. Effects of oral L-arginine on the foetal condition and neonatal outcome in preeclampsia: a preliminary report. Basic Clin Pharmacol Toxicol. 2006;99(2):146-52. doi: 10.1111/j.1742-7843.2006.pto_468.x. PubMed PMID: 16918716.
39. Nakano S, Noguchi T, Takekoshi H, Suzuki G, Nakano M. Maternal-fetal distribution and transfer of dioxins in pregnant women in Japan, and attempts to reduce maternal transfer with Chlorella (Chlorella pyrenoidosa) supplements. Chemosphere. 2005;61(9):1244-55. Epub 20050627. doi: 10.1016/j.chemosphere.2005.03.080. PubMed PMID: 15985279.
40. Helland IB, Saugstad OD, Smith L, Saarem K, Solvoll K, Ganes T, et al. Similar effects on infants of n-3 and n-6 fatty acids supplementation to pregnant and lactating women. Pediatrics. 2001;108(5):E82. doi: 10.1542/peds.108.5.e82. PubMed PMID: 11694666.
41. Awe O, Sinkway JM, Chow RP, Wagener Q, Schulz EV, Yu JY, et al. Differential regulation of a placental SAM68 and sFLT1 gene pathway and the relevance to maternal vitamin D sufficiency. Pregnancy Hypertens. 2020;22:196-203. Epub 20200912. doi: 10.1016/j.preghy.2020.09.004. PubMed PMID: 33068876; PubMed Central PMCID: PMC7688503.
42. Park H, Wood MR, Malysheva OV, Jones S, Mehta S, Brannon PM, et al. Placental vitamin D metabolism and its associations with circulating vitamin D metabolites in pregnant women. Am J Clin Nutr. 2017;106(6):1439-48. Epub 20171011. doi: 10.3945/ajcn.117.153429. PubMed PMID: 29021285; PubMed Central PMCID: PMC5698837.
43. Darling AM, Mugusi FM, Etheredge AJ, Gunaratna NS, Abioye AI, Aboud S, et al. Vitamin A and Zinc Supplementation Among Pregnant Women to Prevent Placental Malaria: A Randomized, Double-Blind, Placebo-Controlled Trial in Tanzania. Am J Trop Med Hyg. 2017;96(4):826-34. Epub 20170123. doi: 10.4269/ajtmh.16-0599. PubMed PMID: 28115667; PubMed Central PMCID: PMC5392628.
44. Johnston PC, McCance DR, Holmes VA, Young IS, McGinty A. Placental antioxidant enzyme status and lipid peroxidation in pregnant women with type 1 diabetes: The effect of vitamin

C and E supplementation. J Diabetes Complications. 2016;30(1):109-14. Epub 20151009. doi: 10.1016/j.jdiacomp.2015.10.001. PubMed PMID: 26597598.
45. Owens S, Gulati R, Fulford AJ, Sosseh F, Denison FC, Brabin BJ, et al. Periconceptional multiple-micronutrient supplementation and placental function in rural Gambian women: a double-blind, randomized, placebo-controlled trial. Am J Clin Nutr. 2015;102(6):1450-9. Epub 20151111. doi: 10.3945/ajcn.113.072413. PubMed PMID: 26561613; PubMed Central PMCID: PMC4658455.
46. Jiang X, Yan J, West AA, Perry CA, Malysheva OV, Devapatla S, et al. Maternal choline intake alters the epigenetic state of fetal cortisol-regulating genes in humans. FASEB J. 2012;26(8):3563-74. Epub 20120501. doi: 10.1096/fj.12-207894. PubMed PMID: 22549509.
47. Mercer BM, Abdelrahim A, Moore RM, Novak J, Kumar D, Mansour JM, et al. The impact of vitamin C supplementation in pregnancy and in vitro upon fetal membrane strength and remodeling. Reprod Sci. 2010;17(7):685-95. doi: 10.1177/1933719110368870. PubMed PMID: 20581351; PubMed Central PMCID: PMC2930608.
48. Kaplas N, Isolauri E, Lampi AM, Ojala T, Laitinen K. Dietary counseling and probiotic supplementation during pregnancy modify placental phospholipid fatty acids. Lipids. 2007;42(9):865-70. Epub 20070724. doi: 10.1007/s11745-007-3094-9. PubMed PMID: 17647038.
49. van Eijk AM, Ayisi JG, Slutsker L, Ter Kuile FO, Rosen DH, Otieno JA, et al. Effect of haematinic supplementation and malaria prevention on maternal anaemia and malaria in western Kenya. Trop Med Int Health. 2007;12(3):342-52. doi: 10.1111/j.1365-3156.2006.01787.x. PubMed PMID: 17313505.
50. Larqué E, Krauss-Etschmann S, Campoy C, Hartl D, Linde J, Klingler M, et al. Docosahexaenoic acid supply in pregnancy affects placental expression of fatty acid transport proteins. Am J Clin Nutr. 2006;84(4):853-61. doi: 10.1093/ajcn/84.4.853. PubMed PMID: 17023713.
51. Klingler M, Blaschitz A, Campoy C, Caño A, Molloy AM, Scott JM, et al. The effect of docosahexaenoic acid and folic acid supplementation on placental apoptosis and proliferation. Br J Nutr. 2006;96(1):182-90. doi: 10.1079/bjn20061812. PubMed PMID: 16870008.
52. Cox SE, Staalsoe T, Arthur P, Bulmer JN, Tagbor H, Hviid L, et al. Maternal vitamin A supplementation and immunity to malaria in pregnancy in Ghanaian primigravids. Trop Med Int Health. 2005;10(12):1286-97. doi: 10.1111/j.1365-3156.2005.01515.x. PubMed PMID: 16359410.
53. Pressman EK, Cavanaugh JL, Mingione M, Norkus EP, Woods JR. Effects of maternal antioxidant supplementation on maternal and fetal antioxidant levels: a randomized, double-blind study. Am J Obstet Gynecol. 2003;189(6):1720-5. doi: 10.1016/s0002-9378(03)00858-5. PubMed PMID: 14710104.
